# Primary fixation stability evaluation of pre-bent titanium miniplate configurations in mandibular reconstruction

**DOI:** 10.3389/fbioe.2026.1697119

**Published:** 2026-02-12

**Authors:** Philipp Ruf, Özgür Cebeci, Vincenzo Orassi, Claudius Steffen, Georg N. Duda, Max Heiland, Sara Checa, Carsten Rendenbach

**Affiliations:** 1 Department of Oral and Maxillofacial Surgery, Charité - Universitätsmedizin Berlin, Corporate Member of Freie Universität Berlin and Humboldt Universität zu Berlin, Berlin, Germany; 2 Julius Wolff Institute, Berlin Institute of Health at Charité - Universitätsmedizin Berlin, Berlin, Germany; 3 Institute of Biomechanics, Hamburg University of Technology (TUHH), Hamburg, Germany

**Keywords:** biomechanics, conventional miniplates, conventional reconstruction plate, finite element analysis, intersegmental strain, mandibular reconstruction, pre-bending, stress

## Abstract

Mandibular reconstruction is a commonly performed procedure in maxillofacial surgery with many different possible reconstructive strategies. Generally, modular fixation with miniplates has the advantage of an easier plate removal, mostly possible through an intraoral access. For patient-specific plates, the combination of reconstruction plates in the posterior area with anterior miniplates has been described as biomechanically and clinically beneficial. To our knowledge, conventional miniplates have not been biomechanically assessed in combination with reconstruction plates. Therefore, the present study aimed to evaluate combinations of pre-bent anterior miniplates with a posterior pre-bent reconstruction plate *in silico* as an alternative to pre-bent fixation strategies including only miniplates. The results indicate, that from a biomechanical perspective, the combination of a posterior pre-bent short reconstruction plate with anterior pre-bent miniplates is a viable alternative to fixation strategies including solely pre-bent miniplates. The process of pre-bending did not affect intersegmental strains. Analogously to patient-specific plates, the combination of a posterior pre-bent reconstruction plate with pre-bent anterior miniplates seems a surgically and biomechanically promising alternative to fixation strategies with pre-bent miniplates alone.

## 1 Introduction

Mandibular reconstruction is a challenging procedure, with different reconstructive options available (Rendenbach et al., 2018; Brown et al., 2017; Ritschl et al., 2021b; Kreutzer et al., 2023). Generally, there are two different reconstructive strategies in primary mandibular reconstruction (Knitschke et al., 2022; Rendenbach et al., 2019):

- An osseous flap (mostly fibula free flap) is harvested using pre-operatively produced cutting guides and fixed with pre-produced patient-specific plates
- The flap is fixed with pre- or intraoperatively bent plates

The first approach has the advantage of reduced surgery time and increased precision (Rendenbach et al., 2018; Mascha et al., 2017; Mahendru et al., 2020; Bengur et al., 2025; Marquardt et al., 2025; Tang et al., 2019), while the second approach is very cost-effective, reduces the time-to-surgery and has been associated with higher rates of osseous union (Ritschl et al., 2021a; Rendenbach et al., 2018; Knitschke et al., 2022; Rendenbach et al., 2019).

For both reconstructive strategies, the use of miniplates–that enable intraoral removal due to their modular design–is possible (Kreutzer et al., 2022a; Robey et al., 2008). Plate removal is particularly necessary before dental rehabilitation, since the fixation material often interferes with the dental implants (Kreutzer et al., 2022b). Previous studies on patient-specific plates regarding biomechanical properties, surgical feasibility and clinical outcomes of pre-planned mandibular reconstructions have stated the particular advantages of the combination of a posterior patient-specific titanium reconstruction plate with anterior patient-specific titanium miniplates (Kreutzer et al., 2023; Ruf et al., 2024). Contrastingly, although pre-bent titanium miniplates are regularly used in the clinical practice (Ritschl et al., 2021a; Weitz et al., 2023) and have been assessed *in vitro* and in clinical studies as viable alternative to pre-bent reconstruction plates (Rendenbach et al., 2017; Steffen et al., 2020; Fontana et al., 2016), to our knowledge, the biomechanical properties induced by combinations of pre-bent reconstruction plates and pre-bent miniplates have not been assessed. Recently, a work-flow has been established to simulate the process of pre-bending–crucial to conventional, pre-bent plates–*in silico* and subsequently assess the primary fixation stability of conventional plates (Ruf et al., 2025).

Therefore, the present study aimed to evaluate the primary fixation stability of pre-bent conventional miniplates alone and in combination with a posterior pre-bent titanium reconstruction plate. To account for effects resulting from pre-bending, the pre-bent plates have been compared to same-shaped but patient-specific plates.

## 2 Materials and methods

### 2.1 Finite element model creation

Finite element models of a reconstructed mandible with a one segmental free fibula transplant of the type L were previously developed (Ruf et al., 2022; Boyd et al., 1993). The model geometry was derived from a pre-operative CT scan of a 57-year-old female patient who underwent segmental mandibular resection due to oral squamous cell carcinoma. Image segmentation and meshing were performed using Amira 6.0.1 (Thermo Fisher Scientific, Waltham, MA, United States). The fibula segment measured 5 cm in length and was positioned according to clinical guidelines from the right angle to the right canine region, leaving an 8 cm residual fibula stump at the donor site.

### 2.2 Fixation configurations

**FIGURE 1:**
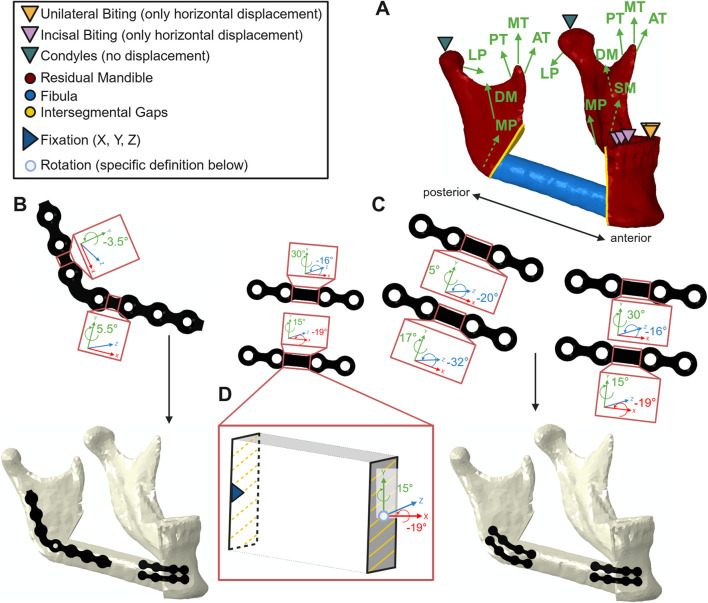
**(A)** Overview over the reconstructed mandible with boundary conditions in the condyles and for unilateral and incisal biting along with participating biting muscles superficial- (SM) and deep masseter (DM), lateral- (LP) and medial pterygoid (MP), anterior- (AT), middle- (MT) and posterior temporalis (PT); **(B)** combination of a short posterior reconstruction plate with anterior miniplates in schematic undeformed configuration including bending angles (top) and in deformed configuration with screws after pre-bending (bottom left); **(C)** plating configuration consisting of four miniplates in schematic undeformed configuration including bending angles (top) and in deformed configuration with screws after pre-bending (bottom); **(D)** exemplary bending segment: fixed posterior midpoint and tied corresponding cross-section area along with anterior mid-point and coupled cross-section area (where rotation and deformation were defined, bottom right) – Created in https://BioRender.com.

Two different fixation approaches were investigated:

1. Four 1.0 mm thick titanium miniplates (Karl Leibinger Medizintechnik GmbH and Co. KG, Tuttlingen, Germany; article number 25–551–04-XX (KLS, 2023a)), two in the front, two in the back (configuration MP, Figure 1C)
2. A combination of a shortened posterior 7-hole 2.0 mm titanium reconstruction plate (Karl Leibinger Medizintechnik GmbH and Co. KG, Tuttlingen, Germany; article number 50–775–26-XX (KLS, 2023b)) and two anterior 1.0 mm thick titanium miniplates (KLS, 2023a) (configuration MIX, Figure 1B).

The abovementioned configurations were investigated in two different states to account for biomechanical effects resulting from pre-bending:

1. Pre-bent configurations including pre-bending process of all plates to fit the reconstructed mandible
2. Same-shaped but patient-specific configurations for comparison, particularly to potentially assign biomechanical observations to the process of pre-bending. The patient-specific design was achieved by deleting plastic stresses in the deformed plates.

The plates were pre-bent in the bridge area in Abaqus CAE 2021 (Dassault Systèmes, Vélizy-Villacoublay, France) to fit the reconstructed mandible. To simulate pre-bending, in each deformed bridge area, a rotation of one bridge cross-section plane around the corresponding cross-section mid-point was defined in local coordinates while the other cross-section plane was fixed (Figure 1D). In a second step, the plates were released to obtain the permanently deformed shape. Subsequently, the pre-bent plates were re-imported along with their plastic strains using the “Predefined Field–Initial State” option in Abaqus CAE 2021 (pre-bent scenario). To design the same-shaped patient-specific plate, the pre-bent plates were re-imported in Abaqus CAE 2021 without their plastic strains. Afterwards, the biting simulation was conducted for all configurations. Monocortical screws of 7 mm in length were used for fixation of the miniplates in all locations and the reconstruction plate on the fibula to prevent damage from the intraosseous vessels. Bicortical screws of varying lengths were used to fix the reconstruction plate on the mandible (8, 7 and 9 mm from posterior to anterior).

### 2.3 Meshing and mesh convergence test

Plate geometries were meshed with quadratic hexahedral elements (C3D20R) to enable the “Predefined Field–Initial State” option in Abaqus CAE 2021 and, thus, the re-import of the pre-bent plates (ABAQUS, 2006; Ruf et al., 2025). A previous study on pre-bent reconstruction plates (same article number as the short reconstruction plate in the present study) included a mesh convergence test resulting in a mesh size of 0.3 mm which was considered valid for the shortened posterior reconstruction plate in the present study (Ruf et al., 2025). For the miniplates, a mesh convergence test with the mesh sizes 0.5 mm, 0.4 mm, 0.3 mm, and 0.25 mm was conducted. A combination of transverse bending (20°) and torsion (30°) was tested. The output parameters were the means of plastic equivalent strain, maximum plastic strain (absolute) and von Mises stress. For all mesh sizes on the miniplates, the relative difference to the next finer mesh was below 5%. To keep consistency, all plates were meshed using a mesh size of 0.3 mm.

The screws and the mandible were meshed in Abaqus CAE 2021 using second-order quadratic tetrahedral elements (Type C3D10). For the screws, a mesh size of 1 mm was chosen. The healing regions of the mandible were meshed with a mesh size of 0.2 mm, based on a mesh convergence study of a previous work (Ruf et al., 2022).

### 2.4 Boundary and loading conditions

Tie constraints were defined between screws and plates and between screws and bone tissue. Biting tasks included unilateral and incisal biting. Analogously to previous *in silico* studies on the reconstructed mandible, the condyles were fixed in all dimensions to simulate the articulation in the mandibular fossa (Ruf et al., 2022; Park et al., 2018). The participating muscles included superficial and deep masseter, anterior, middle and posterior temporalis and medial and lateral pterygoid (Korioth and Hannam, 1994; Korioth, et al., 1992). The superficial masseter was assumed as removed on the resection side and the deep masseter’s activity reduced by 50% on the resection side. Furthermore, maximum muscle forces were scaled to 12.5% to reflect post-operative conditions, resulting in a unilateral bite force of 40 N (Gheibollahi et al., 2021). Muscle forces, fiber directions and fiber activations are presented in Table 1.

**TABLE 1:** Healthy peak muscle forces, a reduction to 12.5% of peak muscle forces, muscle fiber directions, and fiber activations for the muscles involved in the studied biting tasks–unilateral (UNI) and incisal (INC) biting; muscle fiber directions are defined relative to the frontal (XY), transverse (XZ), and sagittal (YZ) planes (Korioth et al., 1992).

Muscle	​	​	Fiber direction				Fiber activation			
	Healthy maximum muscle force (N)	12,5% of maximum muscle force (N)	X		Y	Z	INC		UNI	
	​	​	Right	Left	​	​	Right	Left	Right	Left
Superficial masseter	190,4	23,8	−0,207	0,207	0,884	0,419	0	0,4	0	0,72
Deep masseter	81,6	10,2	−0,546	0,546	0,758	−0,358	0,13	0,26	0,3	0,72
Medial pterygoid	174,8	21,85	0,486	−0,486	0,791	0,373	0,78	0,78	0,6	0,84
Lateral pterygoid	66,9	83,625	0,63	−0,63	−0,174	0,757	0,71	0,71	0,65	0,3
Anterior temporalis	158	19,75	−0,149	0,149	0,988	0,044	0,08	0,08	0,58	0,73
Middle temporalis	95,6	11,95	−0,222	0,222	0,837	−0,5	0,06	0,06	0,67	0,66
Posterior temporalis	75,6	9,45	−0,208	0,208	0,474	−0,855	0,04	0,04	0,39	0,59

### 2.5 Material properties

All plates were made of pure titanium and modeled with isotropic, linear elastic properties up to the yield point and with isotropic, linear plastic behavior beyond the yield point (KLS, 2023b; KLS, 2023a; MatWeb, 2024a). The yield point (defined by the yield stress of 340 MPa and the Young’s modulus of 102 GPa) and the breaking point (defined by elongation at break of 28% and the ultimate stress of 430 MPa) were interpolated to obtain a curve of true stress and plastic strain (MatWeb, 2024a; Ruf et al., 2025). The screws were assumed as isotropic, linear elastic and made of titanium alloy Ti6Al4V (KLS, 2023b; KLS, 2023a; MatWeb, 2024b). Cortical bone was assigned anisotropic, linear-elastic properties (Lefèvre et al., 2015; Rho, 1996; Schwartz-Dabney and Dechow, 2003), while trabecular bone, dentin and granulation tissue were modeled as isotropic and linear elastic (Lovald et al., 2009; Lakatos et al., 2014; Leong and Morgan, 2008). The linear elastic material properties are presented in Table 2.

**TABLE 2:** Anisotropic and isotropic material properties for bone, intersegmental gaps, and fixation materials. 1: longitudinal (along the local bone orientation); 2: tangential; 3: transverse.

Material	Symphysis	Body	Angle	Ramus	Condyle	Coronoid	Fibula cortical	Dentin	Mandible trabecular	Granulation tissue	Ti-6AI-4V	Titanium
E1 (GPa)	20,5	21,7	23,8	24,6	23,5	28	28	17,6	0,3	0,001	114	102
E2 (GPa)	16,4	17,8	19	18,4	17,9	17,5	17,7	17,6	0,3	0,001	114	102
E3 (GPa)	12,1	12,7	12,8	13	12,7	14	17,7	17,6	0,3	0,001	114	102
Nu12	0,34	0,34	0,3	0,28	0,24	0,23	0,237	0,34	0,3	0,3	0,33	0,34
Nu23	0,22	0,2	0,22	0,23	0,25	0,28	0,42	0,34	0,3	0,3	0,33	0,34
Nu13	0,43	0,45	0,41	0,38	0,32	0,28	0,231	0,34	0,3	0,3	0,33	0,34
G12 (GPa)	6,9	7,5	7,6	7,4	7,2	7,2	4,7	6,6	0,115	0,000385	44	38
G23 (GPa)	4,8	5,1	5	5	5,2	5,3	3,6	6,6	0,115	0,000385	44	38
G13 (GPa)	5,3	5,5	5,5	5,4	5,5	5,8	4,7	6,6	0,115	0,000385	44	38

### 2.6 Output evaluation

Mechanical principal strains in the healing regions were evaluated as predictor of bone formation. Strains exceeding 500 µstrain were included in the analysis and outliers were identified using the ROUT method with Q = 0.1% and subsequently removed before percentile calculation (Motulsky and Brown, 2006). Through the exclusion operation, small strain values were eliminated, particularly in osteotomy elements away from the direct interface of different-sized mandible and fibula (Ruf et al., 2022). The strain distributions were quantified in each specific scenario with the percentiles 0.25, 0.5 (median), 0.75 and 1 (highest value after outlier identification) to numerically account for strain distributions. Peaks in von Mises stresses were evaluated to assess the risk of material failure. Stress singularities, possibly occurring due to kinematic constraints during bending or application of tie constraints between different sized meshes, were mitigated by excluding the top 0.03% of values and averaging the top 10 of the remaining values (Ruf et al., 2025).

## 3 Results

### 3.1 Biting forces and strain distribution

Predicted bite forces were between 9 and 10 N for incisal biting and between 38 and 40 N under unilateral biting.

**FIGURE 2:**
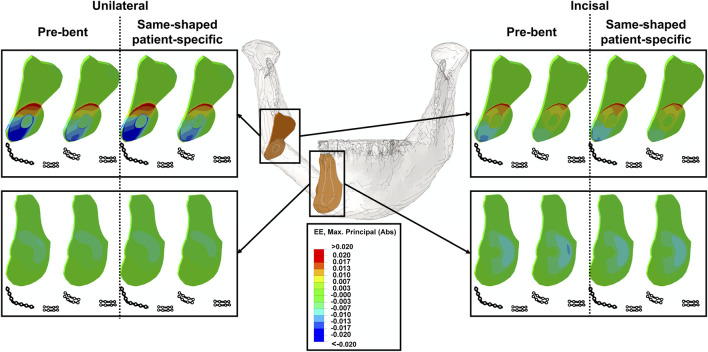
Strain contour plots of the anterior (bottom) and posterior (top) intersegmental gaps for all investigated scenarios including miniplates and a posterior reconstruction plate, unilateral and incisal biting and pre-bent and same-shaped patient-specific plating systems.

All contour plots of the intersegmental strains are presented in Figure 2. Generally, posterior strains were most stimulated by unilateral biting, whereas, anterior strains were more stimulated by incisal biting (Figure 2).

In the posterior area, local application of a reconstruction plate increased intersegmental strains compared to local miniplate fixation under unilateral and incisal biting (Tables 3, 4). However, pre-bending only had a marginal influence on local posterior intersegmental strain levels, in both biting tasks (Tables 3, 4).

**TABLE 3:** Unilateral biting induced posterior intersegmental strain percentiles for the plating configurations combining a posterior reconstruction plate with anterior miniplates (MIX) and the configuration with miniplates alone (MP) in pre-bent and same-shaped patient-specific states.

Unilateral biting posterior percentiles	Pre-bent MIX	Same-shaped patient-specific MIX	Pre-bent MP	Same-shaped patient-specific MP
0.25	0.4%	0.4%	0.4%	0.3%
0.5	0.9%	0.9%	0.5%	0.5%
0.75	1.3%	1.3%	0.9%	0.9%
1	4.1%	4.1%	2.6%	2.6%

**TABLE 4:** Incisal biting induced posterior intersegmental strain percentiles for the plating configurations combining a posterior reconstruction plate with anterior miniplates (MIX) and the configuration with miniplates alone (MP) in pre-bent and same-shaped patient-specific states.

Incisal biting posterior percentiles	Pre-bent MIX	Same-shaped patient-specific MIX	Pre-bent MP	Same-shaped patient-specific MP
0.25	0.3%	0.3%	0.2%	0.2%
0.5	0.5%	0.5%	0.3%	0.3%
0.75	0.8%	0.8%	0.5%	0.5%
1	2.4%	2.3%	1.5%	1.5%

The anterior intersegmental strains under local miniplate fixation were not influenced by the posterior plating system (short reconstruction plate or miniplates) during unilateral and incisal biting (Tables 5, 6). Similar to the posterior area, in the anterior region, intersegmental strains were not determined by the pre-bending process but rather influenced by the biting task (Tables 5, 6). In consequence, in both intersegmental gaps, the strains were not influenced by the process of pre-bending.

**TABLE 5:** Unilateral biting induced anterior intersegmental strain percentiles for the plating configurations combining a posterior reconstruction plate with anterior miniplates (MIX) and the configuration with miniplates alone (MP) in pre-bent and same-shaped patient-specific states.

Unilateral biting anterior percentiles	Pre-bent MIX	Same-shaped patient-specific MIX	Pre-bent MP	Same-shaped patient-specific MP
0.25	0.1%	0.1%	0.2%	0.2%
0.5	0.2%	0.2%	0.2%	0.2%
0.75	0.4%	0.4%	0.4%	0.3%
1	1%	0.9%	1%	0.9%

**TABLE 6:** Incisal biting induced anterior intersegmental strain percentiles for the plating configurations combining a posterior reconstruction plate with anterior miniplates (MIX) and the configuration with miniplates alone (MP) in pre-bent and same-shaped patient-specific states.

Incisal biting anterior percentiles	Pre-bent MIX	Same-shaped patient-specific MIX	Pre-bent MP	Same-shaped patient-specific MP
0.25	0.2%	0.1%	0.2%	0.2%
0.5	0.3%	0.3%	0.3%	0.3%
0.75	0.6%	0.6%	0.6%	0.6%
1	1.3%	1.3%	1.4%	1.3%

### 3.2 Stress distribution in miniplates

Peak stresses within the pre-bent plates are shown in Table 7. Pre-bending induced higher stresses in the miniplates compared with the reconstruction plate, with peak von Mises stresses within the plastic phase of titanium, exceeding the yield stress of 340 MPa. This was associated with the miniplates experiencing maximum bending angles of 32°, in multiple directions (Figure 1). In contrast, in the posterior reconstruction plate, where the maximum deformation angle was 5.5°, the peak stress after the bending and release steps was approximately 305 MPa, in the elastic phase of titanium.

**TABLE 7:** Peak von mises stresses in the pre-bent plates–combination of a posterior reconstruction plate with two anterior miniplates (MIX) and fixation with miniplates alone–in MPa including the scenarios of the pre-bent plates before biting, and the pre-bent plates after unilateral (UNI) and incisal biting (INC).

Scenario	Posterior		Anterior	
​	Inferior	Superior	Inferior	Superior
Pre-bent MIX before biting	305.44		359.02	374.38
Pre-bent MIX (UNI)	312.6		301.01	339.87
Pre-bent MIX (INC)	308.61		301.08	340.89
Pre-bent miniplates before biting	371.45	321.53	359.02	374.38
Pre-bent miniplates (UNI)	349.31	321.95	302.17	339.96
Pre-bent miniplates (INC)	346.84	323.01	301.72	340.84

In the plates pre-stressed through the bending process beyond the yield point, a moderate decrease in von Mises stress through the biting task performance was observed. In contrast, within the plates stressed below the yield stress, the peak stress slightly increased due to the biting simulation.

The peak von Mises stresses of the same-shaped patient-specific plates–moderate elastic stresses with a maximum under 60 MPa–are presented within Table 8. Within those plates, higher peak stresses were observed in the posterior plates and particularly in the posterior miniplates compared to the posterior reconstruction plate. The stresses in the anterior miniplates were approximately 1/3 of the peak von Mises stresses in the posterior plates. Unilateral biting induced higher peak stresses in comparison to incisal biting.

**TABLE 8:** Peak von mises stresses in the same-shaped patient-specific configurations–combination of a posterior reconstruction plate with two anterior miniplates (MIX) and fixation with miniplates alone - in MPa after unilateral (UNI) and incisal biting (INC).

Scenario	Posterior		Anterior	
​	Inferior	Superior	Inferior	Superior
Same-shaped patient-specific MIX (UNI)	42.41		14.06	16.26
Same-shaped patient-specific miniplates (UNI)	58.28	53.14	15.16	13.42
Same-shaped patient-specific MIX (INC)	22.3		13.9	13.15
Same-shaped patient-specific miniplates (INC)	33.72	33.64	11.1	11.36

## 4 Discussion

Mandibular reconstruction is a commonly performed surgical intervention in the maxillofacial field, mostly with a fibula free flap, but with different plating approaches of pre-bent or patient-specific nature (Brown et al., 2017; Weitz et al., 2023; Kreutzer et al., 2023). While patient-specific plates have been extensively biomechanically investigated and this resulted in the clinically preferred combination of a posterior titanium reconstruction plate with two anterior miniplates (Kreutzer et al., 2023; Ruf et al., 2022; Ruf et al., 2024), pre-bent miniplates have primarily been investigated in configurations without reconstruction plates *in vitro* and in clinical studies (Steffen et al., 2020; Rendenbach et al., 2017; Ritschl et al., 2021a). Since recently, a work-flow has been established to assess the primary fixation stability of pre-bent reconstruction plates *in silico* (Ruf et al., 2025), the present study aimed to extend this workflow to pre-bent miniplates to evaluate alternative configurations of pre-bent miniplates, particularly in combination with a short posterior reconstruction plate. The results indicate that the combination of a posterior pre-bent reconstruction plate with two parallel anterior pre-bent miniplates is a viable alternative to a fixation with four titanium miniplates. The process of pre-bending did not influence the intersegmental strains.

Mechanical stimuli, particularly interfragmentary strains, have been shown to influence bone formation in the healing callus (Claes, 2017; Claes and Heigele, 1999; Duda et al., 2023). While specific ranges of mechanical stimuli have been identified as beneficial for bone healing in long bones (Claes and Heigele, 1999), the extent to which intersegmental strains promote healing in the mandible remains unclear. However, a previous study linked improved healing outcomes under pre-bent reconstruction plate fixation to higher levels of intersegmental strain compared to clinically used patient-specific reconstruction plates (Ruf et al., 2025). Additionally, the process of pre-bending was shown not to be causal for this observation (Ruf et al., 2025).

The present study confirms this: pre-bent miniplate configurations and the same-shaped patient-specific miniplate configurations resulted in similar intersegmental strains. Moreover, the configuration combining a posterior reconstruction plate with anterior miniplates maximized strain levels in both healing regions to levels comparable to those reported for pre-bent reconstruction plates previously associated with successful bone healing outcomes (Rendenbach et al., 2019; Knitschke et al., 2022; Ruf et al., 2025). Based on these biomechanical findings, a similarly successful healing response for the combination of a posterior conventional reconstruction plate and anterior miniplates could be expected due to the similar mechanical environment. Indeed, previous clinical studies have reported no differences in healing outcomes when comparing pre-bent reconstruction plates and miniplates (Robey et al., 2008; Zavattero et al., 2014).

However, in the posterior region, intersegmental strains were reduced under local miniplate fixation compared to local reconstruction plate fixation, matching the results of previous studies examining patient-specific reconstruction plates and miniplates (Ruf et al., 2022; Ruf et al., 2024). Thus, a posterior reconstruction plate fixation might optimize the posterior healing outcome in comparison to posterior miniplates in the present one-segmental reconstruction situation. Supporting this, Kreutzer et al. (2023) demonstrated improved bone formation using the hybrid approach with patient-specific posterior reconstruction plates and anterior patient-specific miniplates.

While patient-specific plates experienced moderate elastic stresses with a maximum of 58 MPa, the pre-bent posterior reconstruction plate experienced stresses in the upper elastic range greater than 300 MPa and the pre-bent miniplates partly experienced plastic stresses beyond 340 MPa. In all configurations–pre-bent or patient-specific - posterior miniplates exhibited higher in-plate stresses than the posterior reconstruction plates. To facilitate the pre-bending process, the commercially available reconstruction plate is pre-manufactured to fit the anatomical shape of the mandibular angle (KLS, 2023b), which made a maximum bending angle of only 5.5° necessary. In contrast, the straightly shaped posterior miniplates experienced bending angles of up to 32° and in multiple directions (KLS, 2023a). The greater bending angles might explain the higher in-plate stress values in the posterior pre-bent miniplates in comparison to the posterior short pre-bent reconstruction plate. In the patient-specific configurations without pre-bending, the larger dimensions of the reconstruction plates have been named as causal for reduced in-plate stresses in comparison to miniplates (Si-Myung et al., 2016; Ruf et al., 2025). This interpretation is equally suitable to explain the greater elastic stresses of up to 58 MPa in the posterior patient-specific miniplates in comparison to 42 MPa in the patient-specific posterior reconstruction plate in the present study.

Previous studies investigating one singular pre-bent reconstruction plate without miniplates as fixation strategy for all osteotomies in a mandibular reconstruction found maximum plastic stresses of above 460 MPa (Ruf et al., 2025; Si-Myung et al., 2016). In contrast, in the present study, the short reconstruction plate only experienced a maximum stress in the elastic phase of 310 MPa. This is likely due to the abovementioned small bending angles (maximum of 5.5°) applied to the posterior reconstruction plate within the present study. In the previous studies on pre-bent reconstruction plates, the bending angles were greater because the reconstruction plate was pre-bent also in the anterior area, where commercially available reconstruction plates are not always produced as anatomically fitting (Ruf et al., 2025; Si-Myung et al., 2016; KLS, 2023b). Subsequently, greater bending angles of up to 50° have been reported for reconstruction plates in the anterior areas (Si-Myung et al., 2016; Ruf et al., 2025). At very high bending angles, the thickness of the plate has been reported to increase the in-plate stress when comparing plates with varying thicknesses under the same bending angles (Si-Myung et al., 2016). This explains the reduced maximum stress of 375 MPa in the pre-bent anterior miniplates in the present study–compared to stress values above 460 MPa for reconstruction plates in the anterior areas in a previous study (Ruf et al., 2025). This observation indicates that smaller miniplates might be more stress-resistant in regions where large bending angles are required–in mandibular reconstruction particularly the anterior mandibular region (Si-Myung et al., 2016). Therefore, the combination of a pre-bent reconstruction plate requiring only small bending angles in the posterior area with anterior miniplates–suitable for greater bending angles (Si-Myung et al., 2016) – seems as a suitable configuration from a biomechanical perspective.

But also from a clinical perspective, this combination seems reasonable, since, before dental rehabilitation, particularly the plates in the anterior tooth-bearing region need to be extracted to avoid interference of the screws with the dental implants (Kreutzer et al., 2022b). Outpatient, intraoral plate removal is often only feasible with modularly designed miniplates in the anterior region (Kreutzer et al., 2022a; Kreutzer et al., 2022b). The posterior reconstruction plate often does not require plate extraction because it does not interfere with the dental implants (Kreutzer et al., 2023).

Consequently, combining anterior pre-bent miniplates with a posterior short pre-bent reconstruction plate seems a suitable alternative to fixation configurations with pre-bent miniplates alone to ensure mechanical integrity while facilitating dental rehabilitation. To reduce all in-plate stresses to a moderate elastic level, patient-specific plate design is a valuable tool.

Within the present study, one patient was chosen as example case, which can be considered a limitation regarding generalization of the biomechanical results. However, this case was selected out of a larger cohort to be representative for the clinical indication of mandibular reconstruction. Furthermore, analyzing just one patient case allowed to reduce the risk of anatomical variability influencing the mechanical results. Future studies should investigate the impact of anatomical factors and validate the findings in larger patient cohorts. Nevertheless, the results of this study are consistent with previous works particularly regarding stress levels in pre-bent and same-shaped patient-specific plates (Si-Myung et al., 2016; Ruf et al., 2025). Furthermore, fatigue implications could be considered particularly for pre-bent plates due to cyclic loading in the plastic phase caused by repeated biting. In this context, *in vitro* studies have been previously demonstrated to reliably test fatigue in pre-bent plates (Steffen et al., 2020; Rendenbach et al., 2017). In contrast, using the *in silico* study design of the present study, it is not feasible to account for fatigue. However, it was not an end point of the present study to test fatigue. In contrast, it was the aim to assess the primary fixation stability of pre-bent miniplates alone and in combination with posterior pre-bent reconstruction plates. However, future studies could evaluate the combination of pre-bent posterior reconstruction plates and pre-bent posterior miniplates *in vitro* to account for fatigue.

To conclude, the combination of a pre-bent posterior reconstruction plate with anterior pre-bent miniplates offers both mechanical and clinical advantages in comparison to plating configurations with pre-bent miniplates alone in the chosen one-segmental reconstruction situation. Therefore, it could be considered a clinical alternative. Using patient-specific plates, the plate-failure risk could be reduced by ensuring peak stresses to be in the elastic phase. The pre-bending process itself did not result in differences in intersegmental strain.

## Data Availability

The raw data supporting the conclusions of this article will be made available by the authors, without undue reservation.
